# Tobacco smoking and the risk of gallbladder disease

**DOI:** 10.1007/s10654-016-0124-z

**Published:** 2016-02-22

**Authors:** Dagfinn Aune, Lars J. Vatten, Paolo Boffetta

**Affiliations:** 1 Department of Public Health and General Practice, Faculty of Medicine, Norwegian University of Science and Technology, Trondheim, Norway; 2 Department of Epidemiology and Biostatistics, School of Public Health, Imperial College London, St. Mary’s Campus, Norfolk Place, Paddington, London, W2 1PG UK; 3 Institute of Translational Epidemiology and Tisch Cancer Institute, Icahn School of Medicine at Mount Sinai, New York, NY USA

**Keywords:** Tobacco smoking, Gallstones, Cholelithiasis, Cholecystectomy, Gallbladder disease, Cohort studies, Meta-analysis

## Abstract

Tobacco smoking has been inconsistently associated with gallbladder disease risk. To clarify the association we conducted a systematic review and meta-analysis of cohort studies published on the subject. We searched the PubMed and Embase databases for studies of smoking and gallbladder disease up to January 9th 2015. Prospective studies were included if they reported relative risk estimates and 95 % confidence intervals of gallbladder disease associated with current, former or ever smoking and by number of cigarettes per day. Summary relative risks were estimated by use of a random effects model. We identified ten prospective studies including 59,530 gallbladder disease cases among 4,213,482 participants that could be included in the meta-analysis. The summary RR was 1.19 (95 % CI 1.12–1.28, I^2^ = 46.9 %, n = 6) for current smokers, 1.10 (95 % CI 1.07–1.13, I^2^ = 0 %, n = 6) for former smokers and 1.15 (95 % CI 1.13–1.18, I^2^ = 0 %, n = 7) for ever smokers. In the dose–response analysis the summary relative risk was 1.11 (95 % CI 1.08–1.14, I^2^ = 33 %, n = 3) per 10 cigarettes per day and although there was indication of nonlinearity there was a dose-dependent positive association with increasing number of cigarettes smoked per day. The current meta-analysis provides evidence of an increased risk of gallbladder disease associated with tobacco smoking.

**Electronic supplementary material:**

The online version of this article (doi:10.1007/s10654-016-0124-z) contains supplementary material, which is available to authorized users.

## Introduction

Gallbladder disease is a major cause of morbidity in the US and in the Europe. The prevalence of asymptomatic gallbladder disease ranges between 10–30 % within these populations [1], while symptomatic gallbladder disease is less frequent and affects approximately 2 % of the population [2]. Of digestive diseases that require hospitalization gallbladder disease is the most frequent and costly; the economic costs of hospital treatment of gallbladder disease is over 5 billion US dollar per year [3, 4].

Tobacco smoking is believed to affect the hepatobiliary system and has been associated with increased risk of liver [5] and gallbladder cancer [6]. Studies of smoking in relation to gallbladder disease and gallstones risk have, however, been mixed. Several case–control and cross-sectional studies did not find an association [7–11] or even inverse associations [12], while a few did report increased risk [13, 14], although not always significantly so. However, it is possible that such studies may have been affected by recall bias, selection bias or temporal biases, thus it’s difficult to draw conclusions based on these study designs. Prospective studies, which are less prone to such biases, have also been inconsistent with some studies showing no significant association [15, 16], while some larger studies did report a moderate increase in risk associated with tobacco smoking [17–24], and some [17, 20, 21] of these suggested a dose–response relationship with increasing number of cigarettes smoked per day. We conducted a systematic review and meta-analysis of cohort studies on the association between smoking and gallbladder disease with the aim of clarifying whether there is an association, the strength of any potential association and if there is a dose–response relationship between smoking and gallbladder disease.

## Methods

### Search strategy and inclusion criteria

We developed a systematic review protocol for the project. Pubmed and Embase databases were searched up to January 9th 2015 for eligible studies. As part of a larger project on risk factors for gallbladder disease we used wide search terms PubMed search: (body mass index OR BMI OR overweight OR obesity OR anthropometry OR fatness OR body fatness OR abdominal fatness OR abdominal obesity OR waist circumference OR waist-to-hip ratio OR physical activity OR exercise OR sports OR walking OR biking OR running OR fitness OR exercise test OR inactivity OR diabetes OR smoking OR tobacco OR risk factor OR risk factors) AND (gallstones OR gallbladder disease OR cholelithiasis OR cholecystectomy OR cholecystitis). We followed standard criteria for conducting and reporting meta-analyses [25]. In addition, we searched the reference lists of the identified publications for further studies. One reviewer (DA) conducted the initial screening of all the references and two reviewers (DA, LJV) conducted the screening of the potentially eligible studies. Any disagreements were resolved by discussion between the authors.

#### Study selection

We included published prospective studies that investigated the association between smoking and the risk of gallbladder disease, gallstones, or cholecystectomies. Adjusted estimates of the relative risk had to be available with the 95 % CIs in the publication. For the dose–response analysis a quantitative measure of the smoking level had to be provided. We identified ten relevant prospective studies that could be included in the analysis [15–24]. A list of the excluded studies and the reason for exclusion is provided in Supplementary Table 1.

**Table 1:** Prospective studies of smoking and gallbladder disease

First author, publication year, country	Number of participants, number of cases	Study period	Study quality	Smoking exposure	Comparison and quantity	Relative risk (95 % confidence interval)	Adjustment for confounders
Stampfer MJ et al. 1992, USA	90302 women, age 34–59 years: 2122 symptomatic gallstone cases 488 unremoved gallbladder disease	1980–1988, 6.7 years follow-up	6	Smoking status, cholecystectomy	Never	1.00	Age, body mass index, weight change, alcohol, postmenopausal hormone use, parity, energy intake, polyunsaturated fatty acid intake
					Former	1.06 (0.94–1.18)	
					Current, 1–14 cig/day	1.13 (0.94–1.36)	
					15–24	1.21 (1.04–1.42)	
					25–34	1.36 (1.11–1.67)	
					≥35	1.59 (1.24–2.05)	
				Smoking status, unremoved symptomatic gallstones	Never	1.00	
					Former	1.09 (0.89–1.33)	
					Current, 1–14 cig/day	1.06 (0.74–1.50)	
					15–24	0.93 (0.68–1.29)	
					25–34	1.21 (0.81–1.31)	
					≥35	1.30 (0.78–2.16)	
				Smoking status, both endpoints	Never	1.00	
					Former	1.06 (0.96–1.17)	
					Current, 1–14 cig/day	1.10 (0.93–1.29)	
					15–24	1.03 (0.90–1.19)	
					25–34	1.31 (1.09–1.58)	
					≥35	1.51 (1.20–1.89)	
Kato I, 1992, USA	7831 Japanese men, age 45 to ≥65: 471 gallbladder disease	1965–1968–1990, 19.5 years follow-up	7	Smoking status	Never	1.0	Age
					Former	1.1 (0.9–1.5)	
					Current	1.3 (1.0–1.6)	
				Pack-years of cigarettes	Non-smoker	1.0	
					<24.0 pack-years	1.0 (0.8–1.3)	
					24.0–40.0	1.3 (1.0–1.7)	
					>40.0	1.4 (1.1–1.8)	
Murray FE et al. 1994, United Kingdom	46,000 women, age NA: 1087 gallbladder disease cases	1968–1969–1987, 19 years follow-up	6	Smoking status	Non-smokers	1.00	Age, parity, social class at recruitment
					Smokers	1.19 (1.06–1.34)	
Grodstein F et al. 1994, USA	96,211 women, age 25–42 years: 425 gallstone cases	1989–1991, 2 years follow-up	6	Smoking status	Never	1.0	Age, oral contraceptive use, postmenopausal hormone use, parity, alcohol, body mass index, weight change
					Former	1.1 (0.8–1.4)	
					Current	1.3 (1.0–1.7)	
Misciagna G et al. 1996, Italy	1962 men and women, age 30–69 years: 104 gallstone cases	1985–1986–1992–1993, ~ 7 years follow-up	8	Cigarette smoking	No	1.00	Age, sex, body mass index, weight change, years of schooling, use of laxatives, diabetes, wholemeal bread, fish, fried foods, olive oil, wine, coffee
					Yes	2.15 (1.31–3.54)	
Sahi T et al. 1998, USA	16,414 men, age 15–24 years: 268 cases of gallbladder disease	1962–1966–1977, ~ 13 years follow-up	6	Smoking	Never	1.00	Age, calendar year, body mass index, body mass index change between college and 1962/66, physical activity index
					Former	1.28 (0.89–1.85)	
					Current, < 1 pack/day	1.43 (1.00–2.06)	
					≥1 pack/day	1.52 (1.03–2.24)	
Yamada M et al. 2005, Japan	11,982 men and women, age 13–98 years: 1136 gallstone cases	1958–1998, ~23.6 years follow-up	6	Smoking	Never	1.00	Age, sex, city, period, age, radiation dose, drinking
					Ever	1.19 (1.02–1.40)	
Gonzalez-Perez A et al. 2007, United Kingdom	Nested case–control study: 2353 gallbladder disease cases 10000 controls Men and women, age 20–79 years	1996–1996, 0.9 years follow-up	7	Smoking status	Never	1.00	Age, sex, diabetes, alcohol, body mass index, heart failure, hyperlipidemia, hypertension, ischemic heart disease, stroke, osteoarthritis, rheumatoid arthritis
					Former	1.18 (0.99–1.41)	
					Current	1.05 (0.94–1.19)	
Liu B et al. 2009, United Kingdom	1,290,413 women, mean age 56 years: 23989 gallbladder disease cases	1996–2001–2005, 6.1 years follow-up	9	Cigarette smoking	Never	1.00	Age, region of recruitment, socio-economic status, body mass index, alcohol
					Former	1.10 (1.06–1.13)	
					Current, 1–9 cig/d	1.12 (1.05–1.19)	
					10–19	1.23 (1.17–1.28)	
					≥20	1.29 (1.22–1.37)	
Etminan M et al. 2011, USA	2,721,014 women, mean age ~28.4 years: 27,087 cholecystectomies	1997–2009, 0.9 years follow-up	6	Smoking	No	1.00	Age, obesity, diabetes, inflammatory bowel disease, pancreatitis, sickle-cell anemia, statin use, fibrate use, oral contraceptive use
					Yes	2.06 (1.99–2.14)	

#### Data extraction

The following data were extracted from each study: The first author’s last name, publication year, country where the study was conducted, study period, sample size, sex, number of cases, smoking type, cigarettes per day, relative risks and 95 % confidence intervals for the highest versus the lowest level of smoking and variables adjusted for in the analysis. One reviewer extracted the data (DA) and they were checked for accuracy by a second reviewer (LJV). Any disagreements were resolved by discussion.

### Statistical methods

We calculated summary relative risks for the highest versus the lowest level of smoking using the random-effects model by DerSimonian and Laird [26] which takes into account both within and between study variation (heterogeneity). The average of the natural logarithm of the relative risks was estimated and the relative risk from each study was weighted by the inverse of its variance.

To investigate whether the number of cigarettes smoked per day was associated with gallbladder disease we used the method described by Greenland and Longnecker [27] to conduct dose–response analysis by computing study-specific slopes (linear trends) and 95 % confidence intervals from the natural log of the relative risks and confidence intervals across categories of cigarettes per day. The method requires that the distribution of cases and person-years or non-cases and the relative risks with the variance estimates for at least three quantitative exposure categories are known. For studies that did not provide this information, we estimated the distribution of cases and person-years or non-cases based on a method previously described [28]. Studies that did not quantify the number of cigarettes smoked per day were excluded from the dose–response analysis. We assessed a potential nonlinear dose–response relationship between smoking and gallbladder disease using fractional polynomial models. We determined the best fitting second order fractional polynomial regression model, defined as the one with the lowest deviance. A likelihood ratio test was used to assess the difference between the nonlinear and linear models to test for nonlinearity [29].

Heterogeneity between studies was evaluated using Q and I^2^ statistics [30]. All statistical tests were two-sided and *p* < 0.05 considered statistically significant. I^2^-values of 25, 50 and 75 % indicates low, moderate and high heterogeneity, respectively [31]. We conducted main analyses (all studies combined) and stratified by study characteristics such as sample size, number of cases, geographic location, study quality score and by adjustment for confounding factors. Study quality was assessed using the Newcastle-Ottawa scale which ranks the studies on a scale from 0 to 9 based on the selection of the study population, comparability between cases and non-cases and the assessment of the outcome [32].

Publication bias was assessed using Egger’s test [33] and Begg-Mazumdar’s test [34] and with funnel plots, and *p* < 0.10 was considered to indicate possible publication bias as the tests have low power when the number of studies is low. The statistical analyses were conducted using the software package Stata, version 9.0 software (StataCorp, Texas, US).

## Results

**Fig. 1:**
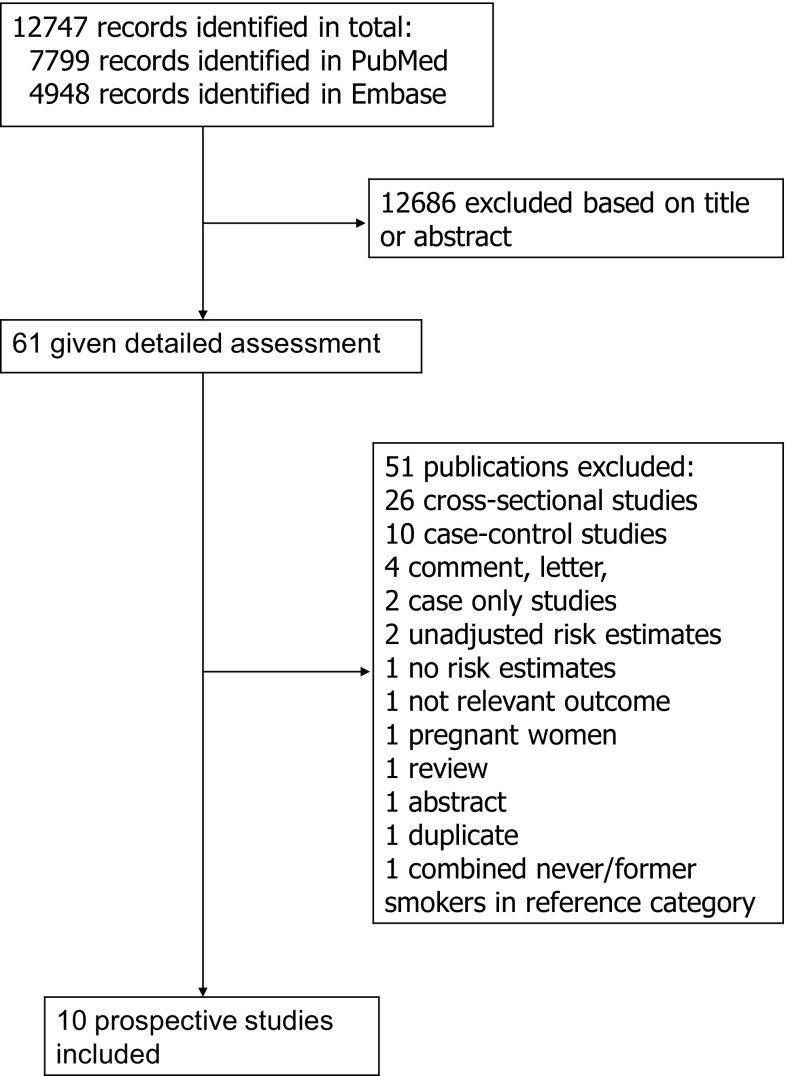
Flow-chart of study selection

Out of a total of 12,747 records identified by the searches, we identified 10 prospective studies [15–24] involving a total of 61,071 cases among 4,344,553 participants that could be included in the analyses of smoking and gallbladder disease (Fig. 1, Table 1). Five of the studies were from North-America, four were from Europe and one was from Asia (Table 1).

### Smoking status

**Fig. 2:**
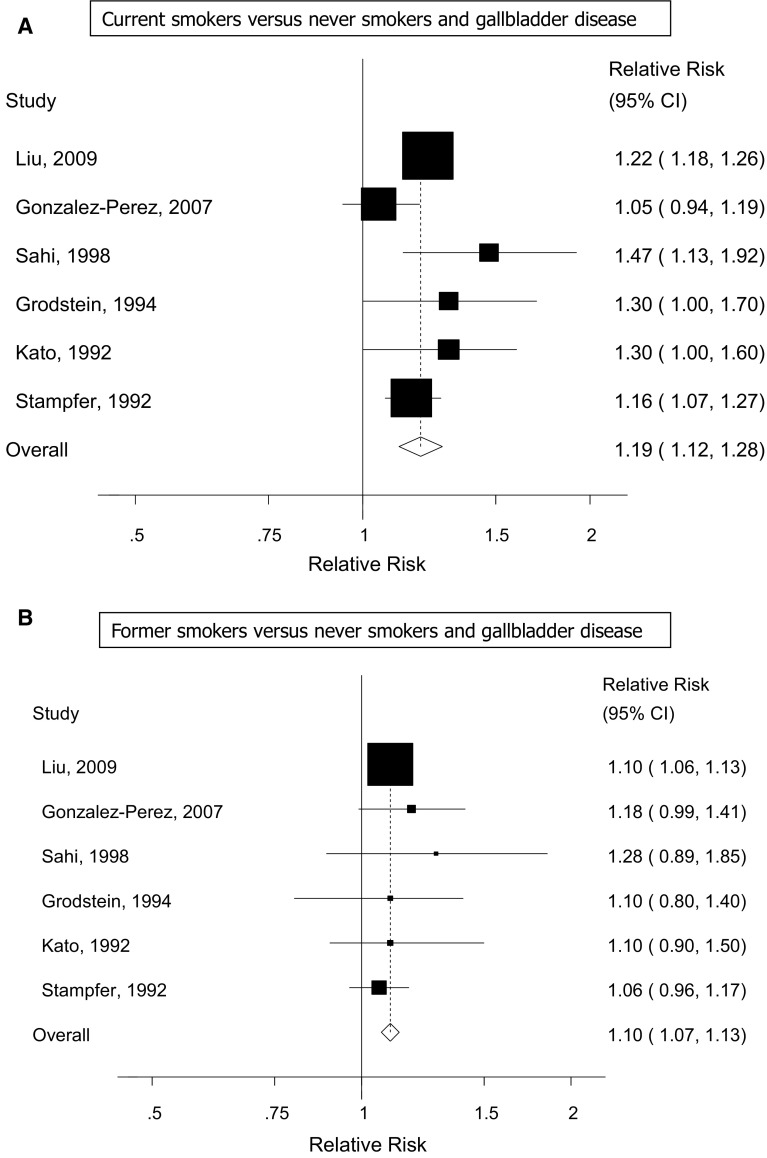
Current smokers (**a**) and former smokers (**b**) versus never smokers and gallbladder disease

**Fig. 3:**
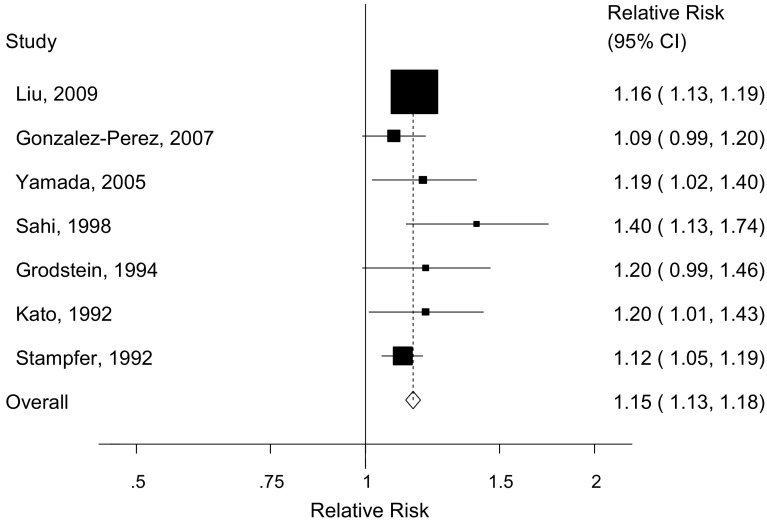
Ever smokers versus never smokers and gallbladder disease

Six cohort studies [16–21] were included in the analysis of current smoking and gallbladder disease and included 30,533 cases among 1,513,524 participants. The summary RR was 1.19 (95 % CI 1.12–1.28, I^2^ = 46.9 %, p_heterogeneity_ = 0.09) (Fig. 2a). Six cohort studies [16–21] were included in the analysis of former smoking and gallbladder disease (30,533 cases and 1,513,524 participants) and the summary RR was 1.10 (95 % CI 1.07–1.13, I^2^ = 0 %, p_heterogeneity_ = 0.88) (Fig. 2b). Seven cohort studies [16–21, 24] were included in the analysis of ever smoking and gallbladder disease (31,669 cases and 1,525,506 participants) and the summary RR was 1.15 (95 % CI 1.13–1.18, I^2^ = 0 %, p_heterogeneity_ = 0.43) (Fig. 3). Three cohort studies [15, 22, 23] with 28,278 cases and 2768976 participants, where the smoking status or the reference category was not clearly defined, were analyzed separately and gave a summary RR of 1.70 (95 % CI 1.09–2.67, I^2^ = 97 %, p_heterogeneity_ < 0.0001) (Supplementary Figure 1). There was no evidence of publication bias in the analysis of current smokers, *p* = 0.98 and *p* = 0.99 with Egger’s test and with Begg’s test respectively, former smokers, *p* = 0.46 and *p* = 0.71, or ever smokers, *p* = 0.58 and *p* = 0.37, respectively, although there was possibly slight asymmetry in the funnel plots (Supplementary Figures 2-4). However, this was driven by one or two outlying studies which did not affect the overall summary estimates.

### Dose–response analyses

**Fig. 4:**
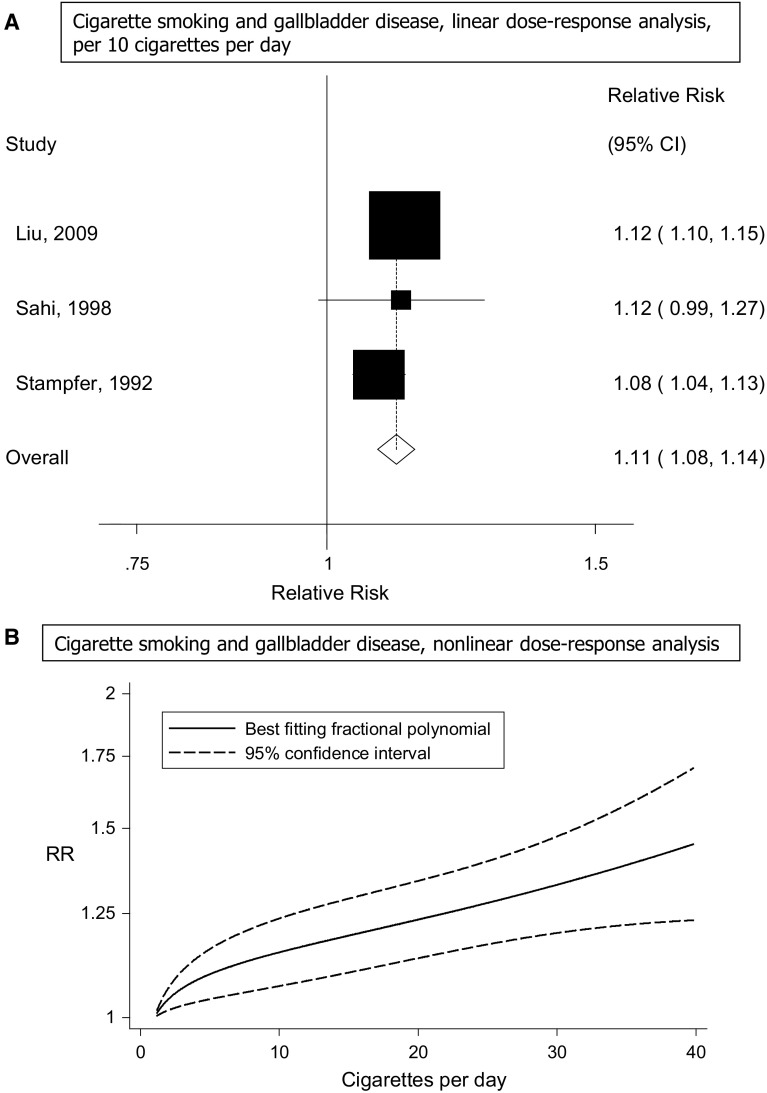
Cigarette smoking and gallbladder disease, linear and nonlinear dose–response analyses

Three cohort studies [17, 20, 21] were included in the dose–response analysis of cigarettes per day and gallbladder disease risk. The summary relative risk was 1.11 (95 % CI 1.08–1.14, I^2^ = 33 %, p_heterogeneity_ = 0.23) per 10 cigarettes per day (Fig. 4a). There was some suggestion of a nonlinear association, p_nonlinearity_ < 0.0001, with a slightly steeper increase in the risk from low levels, but the association appeared to be linear from about 5 cigarettes per day (Fig. 4b).

### Subgroup and sensitivity analyses and cumulative meta-analyses

In subgroup analyses there were positive associations in most strata, defined by study design, gender, outcometype, geographic location, number of cases, study quality and adjustment for confounding factors (Table 2). With meta-regression analyses there was little evidence that the results differed between these subgroups (Table 2). When we conducted sensitivity analyses removing one study at a time, the size of the summary estimates were very similar (Supplementary Figures 5-7). In cumulative meta-analyses, there was little change in the size of the summary RRs over time (Supplementary Figures 8-10), although for former smoking the association became significant in 2007, while for current and ever smoking the associations were significant from the first studies published in 1992.

**Table 2:** Subgroup analyses of smoking and gallbladder disease

	Current smoking					Former smoking					Ever smoking				
	*n*	Relative risk (95 % CI)	*I* ^2^ (%)	*P*_h_^a^	*P*_h_^2^	*n*	Relative risk (95 % CI)	*I* ^2^ (%)	*P*_h_^a^	*P*_h_^b^	*n*	Relative risk (95 % CI)	*I* ^2^ (%)	*P*_h_^a^	*P*_h_^b^
All studies	6	1.19 (1.12–1.28)	46.9	0.09		6	1.10 (1.07–1.13)	0	0.88		7	1.15 (1.13–1.18)	0	0.43	
Follow-up															
<10 years	4	1.17 (1.09–1.26)	56.1	0.08	0.20	4	1.10 (1.07–1.13)	0	0.77	0.66	4	1.15 (1.12–1.18)	0	0.48	0.22
≥10 years	2	1.37 (1.15–1.64)	0	0.50		2	1.16 (0.94–1.43)	0	0.51		3	1.24 (1.12–1.37)	0	0.45	
Gender															
Men	2	1.37 (1.15–1.64)	0	0.50	0.05/0.18^d^	2	1.16 (0.94–1.43)	0	0.51	0.78/0.66^d^	2	1.28 (1.10–1.48)	15.9	0.28	0.21/0.25^d^
Women	3	1.21 (1.18–1.25)	0	0.49		3	1.10 (1.06–1.13)	0	0.78		3	1.15 (1.13–1.18)	0	0.55	
Men and women	1	1.05 (0.94–1.19)				1	1.18 (0.99–1.41)				2	1.12 (1.03–1.21)	0	0.35	
Study design															
Cohort	5	1.22 (1.18–1.25)	0	0.45	0.08	5	1.10 (1.07–1.13)	0	0.88	0.47	6	1.16 (1.13–1.19)	0	0.48	0.28
Nested case–control study	1	1.05 (0.94–1.19)				1	1.18 (0.99–1.41)				1	1.09 (0.99–1.20)			
Outcometype															
Gallbladder disease	5	1.19 (1.10–1.28)	56.1	0.06	0.52	5	1.21 (1.18–1.25)	0	0.43	0.05	5	1.15 (1.10–1.20)	29.3	0.23	0.65
Gallstones	2	1.16 (1.01–1.33)	1.0	0.32		2	1.09 (0.93–1.29)	0	0.96		3	1.15 (1.05–1.25)	0	0.66	
Cholecystectomy	1	1.27 (1.15–1.39)				1	1.06 (0.94–1.18)				1	1.18 (1.09–1.27)			
Geographic location															
Europe	2	1.14 (0.99–1.32)	82.7	0.02	0.38	2	1.10 (1.07–1.14)	0	0.44	0.67	2	1.14 (1.09–1.20)	33.3	0.22	0.63
America	4	1.23 (1.12–1.36)	17.4	0.30		4	1.08 (0.99–1.17)	0	0.80		4	1.18 (1.08–1.28)	30.1	0.23	
Asia	0					0					1	1.19 (1.02–1.40)			
Number of cases															
Cases <1000	3	1.35 (1.17–1.56)	0	0.75	0.17	3	1.14 (0.96–1.34)	0	0.77	0.72	3	1.25 (1.12–1.40)	0	0.49	0.21
Cases ≥1000	3	1.16 (1.07–1.25)	69.2	0.04		3	1.10 (1.07–1.13)	0	0.57		4	1.15 (1.12–1.18)	0	0.48	
Study quality															
0–3 stars					0.55	0				0.65					0.83
4–6 stars	3	1.24 (1.08–1.43)	37.4	0.20		3	1.08 (0.98–1.18)	0	0.61		4	1.18 (1.08–1.28)	29.2	0.24	
7–9 stars	3	1.17 (1.05–1.31)	67.5	0.05		3	1.10 (1.07–1.14)	0	0.75		3	1.16 (1.13–1.18)	0	0.43	
*Adjustment for confounding factors* ^c^															
Age															
Yes	6	1.19 (1.12–1.28)	46.9	0.09	NC	6	1.10 (1.07–1.13)	0	0.88	NC	7	1.15 (1.13–1.18)	0	0.43	NC
No	0					0					0				
Alcohol															
Yes	4	1.17 (1.09–1.26)	56.1	0.08	0.20	3	1.10 (1.07–1.13)	0	0.77	0.66	5	1.15 (1.13–1.18)	0	0.62	0.20
No	2	1.37 (1.15–1.64)	0	0.50		2	1.16 (0.94–1.43)	0	0.51		2	1.28 (1.10–1.48)	15.9	0.28	
BMI															
Yes	5	1.19 (1.10–1.28)	55.6	0.06	0.59	5	1.10 (1.07–1.13)	0	0.77	0.99	5	1.15 (1.10–1.20)	28.8	0.23	0.61
No	1	1.30 (1.00–1.60)				1	1.10 (0.90–1.50)				2	1.19 (1.06–1.34)	0	0.94	
Weight change or BMI change															
Yes	3	1.24 (1.08–1.43)	37.4	0.20	0.55	3	1.08 (0.98–1.18)	0	0.61	0.65	3	1.19 (1.06–1.35)	50.4	0.13	0.83
No	3	1.17 (1.05–1.31)	67.5	0.05		3	1.10 (1.07–1.14)	0	0.75		4	1.16 (1.13–1.19)	0	0.61	
Hormone replacement therapy															
Yes	2	1.17 (1.08–1.27)	0	0.42	0.74	2	1.06 (0.97–1.17)	0	0.81	0.56	2	1.13 (1.06–1.20)	0	0.51	0.50
No	2	1.14 (0.99–1.32)	82.7	0.02		2	1.10 (1.07–1.14)	0	0.44		3	1.16 (1.13–1.18)	0	0.44	
Oral contraceptive use															
Yes	1	1.30 (1.00–1.70)			0.54	1	1.10 (0.80–1.40)			0.99	1	1.20 (0.99–1.46)			0.69
No	3	1.16 (1.07–1.25)	69.2	0.04		3	1.10 (1.07–1.13)	0	0.57		4	1.15 (1.12–1.18)	0	0.48	
Parity															
Yes	2	1.17 (1.08–1.27)	0	0.42	0.74	2	1.06 (0.97–1.17)	0	0.81	0.56	2	1.13 (1.06–1.20)	0	0.51	0.50
No	2	1.14 (0.99–1.32)	82.7	0.02		2	1.10 (1.07–1.14)	0	0.44		3	1.16 (1.13–1.18)	0	0.44	
Physical activity															
Yes	1	1.47 (1.13–1.92)			0.21	1	1.28 (0.89–1.85)			0.46	1	1.40 (1.13–1.74)			0.14
No	5	1.18 (1.11–1.26)	44.9	0.12		5	1.10 (1.07–1.13)	0	0.89		6	1.15 (1.13–1.18)	0	0.72	

*n* denotes the number of studies

^a^P for heterogeneity within each subgroup

^b^P for heterogeneity between subgroups with meta-regression analysis

^c^Number of studies may not add up to the total because some studies did not report the information or the subgroup analysis may not apply to some studies (e.g. subgroup analyses of HRT, OC use and parity are restricted to studies including women)

^d^P for heterogeneity between men and women (excluding studies with both genders)

## Discussion

To our knowledge this is the first meta-analysis of observational studies of smoking and risk of gallbladder disease and our results confirm a 19 % increased relative risk among current smokers, with a dose–response relationship of increasing risk with increasing number of cigarettes smoked per day. In addition, a 10 and 15 % increase in the relative risk was observed for former and ever smokers as well.

Little is known about the biological mechanisms that could explain the adverse effect of smoking on risk of gallbladder disease. Smoking has been shown to increase the risk of type 2 diabetes [35] which is a risk factor for gallbladder disease [36]. Cigarette smoking has also been associated with increased risk of gallbladder cancer [6], although the exact mechanism is not known. In addition, smoking may increase gallbladder disease risk by reducing plasma high density lipoprotein cholesterol concentrations [37] as higher levels of HDL cholesterol are associated with lower gallbladder disease risk [38]. Tobacco smoke contains several dozens of toxic chemicals that may have detrimental effects on the gallbladder by as yet unidentified mechanisms. Further studies are needed to clarify the mechanism(s) that may explain the increased risk of gallbladder disease among smokers.

The present systematic review and meta-analysis has some limitations that need to be discussed. The number of studies included was moderate and some studies could not be included in the dose–response analysis because only smoking status and not results for number of cigarettes per day were reported. Further studies should aim to clarify the dose–response relationship between number of cigarettes per day, duration of smoking, and time since quitting smoking in relation to gallbladder disease risk and report sufficient details to be included in future updated dose–response analyses. Many of the included studies adjusted for important confounding factors and the results persisted in subgroup analyses by whether the studies adjusted for body mass index, weight change, alcohol, hormone replacement therapy use and parity, although there were few studies in some of these subgroup analyses. Publication bias is a possibility, but we did not find evidence of such bias with the statistical tests used or by inspection of the funnel plots, although the number of studies was moderate. Strengths of the present meta-analysis include the detailed dose–response, subgroup and sensitivity analyses, and the large sample size providing a robust estimate of the association between smoking and risk of gallbladder disease.

In conclusion, the results from this systematic review and meta-analysis provide further evidence that smoking increases the risk of developing gallbladder disease. Considering the relatively few modifiable risk factors that have been established for gallbladder disease as well as the many other adverse effects of smoking, further efforts to reduce the prevalence of smoking are needed. Any further studies should report more detailed results by intensity and duration of smoking and clarify the impact of smoking cessation on gallbladder disease risk.

## Electronic supplementary material

Below is the link to the electronic supplementary material.

Supplementary material 1 (DOCX 91 kb)
