# Supplementary material for: Tobacco smoking and the risk of gallbladder disease
Source: Eur J Epidemiol. 2016 Feb 22;31:643–53. doi: 10.1007/s10654-016-0124-z (PMC4977331; doi:10.1007/s10654-016-0124-z)
Supplement: Supplementary file 1 — Supplementary material 1 (DOCX 91 kb) [file 10654_2016_124_MOESM1_ESM.docx]

Supplementary Table 1. List of excluded studies and exclusion reason.

| Exclusion reason | Reference number |
| --- | --- |
| Case-control study | (1-10) |
| Case only study | (11;12) |
| Cross-sectional study | (13-39) |
| Duplicate | (40) |
| Gallstones in pregnancy | (41) |
| Letter, comment | (42-45) |
| No risk estimates | (46) |
| Not relevant outcome | (47) |
| Reference category included never and former smokers combined | (48) |
| Review | (49) |
| Unadjusted risk estimates | (50;51) |

Reference List

1. Pastides H, Tzonou A, Trichopoulos D et al. A case-control study of the relationship between smoking, diet, and gallbladder disease. Arch Intern Med 1990;150:1409-12.

2. La VC, Negri E, D'Avanzo B, Franceschi S, Boyle P. Risk factors for gallstone disease requiring surgery. Int J Epidemiol 1991;20:209-15.

3. Rhodes M, Venables CW. Symptomatic gallstones--a disease of non-smokers? Digestion 1991;49:221-6.

4. McMichael AJ, Baghurst PA, Scragg RK. A case-control study of smoking and gallbladder disease: importance of examining time relations. Epidemiology 1992;3:519-22.

5. Bodmer M, Brauchli YB, Krahenbuhl S, Jick SS, Meier CR. Statin use and risk of gallstone disease followed by cholecystectomy. JAMA 2009;302:2001-7.

6. Sachdeva S, Khan Z, Ansari MA, Khalique N, Anees A. Lifestyle and gallstone disease: scope for primary prevention. Indian J Community Med 2011;36:263-7.

7. Bodmer M, Brauchli YB, Jick SS, Meier CR. Diabetes mellitus and the risk of cholecystectomy. Dig Liver Dis 2011;43:742-7.

8. Linos AD, Daras V, Linos DA, Kekis V, Tsoukas MM, Golematis V. Dietary and other risk factors in the aetiology of cholelithiasis: a case control study. HPB Surg 1989;1:221-7.

9. Laakso M, Suhonen M, Julkunen R, Pyorala K. Plasma insulin, serum lipids and lipoproteins in gall stone disease in non-insulin dependent diabetic subjects: a case control study. Gut 1990;31:344-7.

10. Panpimanmas S, Manmee C. Risk factors for gallstone disease in a Thai population. J Epidemiol 2009;19:116-21.

11. Jayanthi V. Pattern of gall stone disease in Madras city, south India--a hospital based survey. J Assoc Physicians India 1996;44:461-4.

12. Weerakoon HTW, Ranasinghe JGS, Navaratna A, Sivakanesan R, Galketiya KB, Rosairo S. Can the type of gallstones be predicted with known possible risk factors?: A comparison between mixed cholesterol and black pigment stones. BMC Gastroenterology 2014;14:88.

13. Jorgensen T, Kay L, Schultz-Larsen K. The epidemiology of gallstones in a 70-year-old Danish population. Scand J Gastroenterol 1990;25:335-40.

14. Chen CY, Lu CL, Huang YS et al. Age is one of the risk factors in developing gallstone disease in Taiwan. Age Ageing 1998;27:437-41.

15. Diehl AK, Stern MP, Ostrower VS, Friedman PC. Prevalence of clinical gallbladder disease in Mexican-American, Anglo, and black women. South Med J 1980;73:438-41, 443.

16. Diehl AK, Haffner SM, Hazuda HP, Stern MP. Coronary risk factors and clinical gallbladder disease: an approach to the prevention of gallstones? Am J Public Health 1987;77:841-5.

17. Jorgensen T. Gall stones in a Danish population. Relation to weight, physical activity, smoking, coffee consumption, and diabetes mellitus. Gut 1989;30:528-34.

18. Mohr GC, Kritz-Silverstein D, Barrett-Connor E. Plasma lipids and gallbladder disease. Am J Epidemiol 1991;134:78-85.

19. Kono S, Shinchi K, Ikeda N, Yanai F, Imanishi K. Prevalence of gallstone disease in relation to smoking, alcohol use, obesity, and glucose tolerance: a study of self-defense officials in Japan. Am J Epidemiol 1992;136:787-94.

20. Kono S, Shinchi K, Todoroki I et al. Gallstone disease among Japanese men in relation to obesity, glucose intolerance, exercise, alcohol use, and smoking. Scand J Gastroenterol 1995;30:372-6.

21. Attili AF, Capocaccia R, Carulli N et al. Factors associated with gallstone disease in the MICOL experience. Multicenter Italian Study on Epidemiology of Cholelithiasis. Hepatology 1997;26:809-18.

22. Martinez de PC, Carballo F, Horcajo P et al. Prevalence and associated factors for gallstone disease: results of a population survey in Spain. J Clin Epidemiol 1997;50:1347-55.

23. Kratzer W, Kachele V, Mason RA et al. Gallstone prevalence in relation to smoking, alcohol, coffee consumption, and nutrition. The Ulm Gallstone Study. Scand J Gastroenterol 1997;32:953-8.

24. Chen CY, Lu CL, Lee PC, Wang SS, Chang FY, Lee SD. The risk factors for gallstone disease among senior citizens: an Oriental study. Hepatogastroenterology 1999;46:1607-12.

25. Sasazuki S, Kono S, Todoroki I et al. Impaired glucose tolerance, diabetes mellitus, and gallstone disease: an extended study of male self-defense officials in Japan. Eur J Epidemiol 1999;15:245-51.

26. Everhart JE, Khare M, Hill M, Maurer KR. Prevalence and ethnic differences in gallbladder disease in the United States. Gastroenterology 1999;117:632-9.

27. Coelho JC, Bonilha R, Pitaki SA et al. Prevalence of gallstones in a Brazilian population. Int Surg 1999;84:25-8.

28. Pacchioni M, Nicoletti C, Caminiti M et al. Association of obesity and type II diabetes mellitus as a risk factor for gallstones. Dig Dis Sci 2000;45:2002-6.

29. Singh V, Trikha B, Nain C, Singh K, Bose S. Epidemiology of gallstone disease in Chandigarh: a community-based study. J Gastroenterol Hepatol 2001;16:560-3.

30. Everhart JE, Yeh F, Lee ET et al. Prevalence of gallbladder disease in American Indian populations: findings from the Strong Heart Study. Hepatology 2002;35:1507-12.

31. Lai SW, Ng KC. Risk factors for gallstone disease in a hospital-based study. South Med J 2002;95:1419-23.

32. Kono S, Eguchi H, Honjo S et al. Cigarette smoking, alcohol use, and gallstone risk in Japanese men. Digestion 2002;65:177-83.

33. Okamoto M, Yamagata Z, Takeda Y, Yoda Y, Kobayashi K, Fujino MA. The relationship between gallbladder disease and smoking and drinking habits in middle-aged Japanese. J Gastroenterol 2002;37:455-62.

34. Sakuta H, Suzuki T. Plasma total homocysteine and gallstone in middle-aged Japanese men. J Gastroenterol 2005;40:1061-4.

35. Escobar V, Oakes SL, Wood R, Becho J, Markides K, Espino DV. Prevalence and characteristics associated with self-reported gall bladder disease in Mexican American elders: results from the Hispanic Established Populations for Epidemiologic Studies in the Elderly (H-EPESE). Aging Clin Exp Res 2009;21:33-7.

36. Friedrich N, Volzke H, Hampe J, Lerch MM, Jorgensen T. Known risk factors do not explain disparities in gallstone prevalence between Denmark and northeast Germany. Am J Gastroenterol 2009;104:89-95.

37. Walcher T, Haenle MM, Mason RA, Koenig W, Imhof A, Kratzer W. The effect of alcohol, tobacco and caffeine consumption and vegetarian diet on gallstone prevalence. Eur J Gastroenterol Hepatol 2010;22:1345-51.

38. Lee Y-C, Wu J-S, Yang Y-C, Cang C-S, Lu F-H, Chang C-J. Hepatitis B and hepatitis C associated with risk of gallstone disease in elderly adults. Journal of the American Geriatrics Society 2014;62:August.

39. Lee Y-C, Wu J-S, Yang Y-C, Chang C-S, Lu F-H, Chang C-J. Moderate to severe, but not mild, nonalcoholic fatty liver disease associated with increased risk of gallstone disease. Scandinavian Journal of Gastroenterology 2014;49:August.

40. Syngal S, Coakley EH, Willett WC, Byers T, Williamson DF, Colditz GA. Long-term weight patterns and risk for cholecystectomy in women. Ann Intern Med 1999;130:471-7.

41. Basso L, McCollum PT, Darling MR, Tocchi A, Tanner WA. A descriptive study of pregnant women with gallstones. Relation to dietary and social habits, education, physical activity, height, and weight. Eur J Epidemiol 1992;8:629-33.

42. Pastides H. Smoking and the risk of gallstones (Reply). Archives of Internal Medicine 1991;151:1991.

43. Math MV. Smoking and risk of gallstone disease. Am J Public Health 1988;78:100.

44. Lowenfels AB. Smoking and the risk of gallstones. Arch Intern Med 1991;151:398.

45. Thijs C, Knipschild P, Leffers P. Risk factors for symptomatic gall bladder disease. Gut 1994;35:1506.

46. Friedman GD, Kannel WB, Dawber TR. The epidemiology of gallbladder disease: observations in the Framingham Study. J Chronic Dis 1966;19:273-92.

47. Ozasa K. Smoking and mortality in the Japan Collaborative Cohort Study for Evaluation of Cancer (JACC). Asian Pac J Cancer Prev 2007;8 Suppl:89-96.

48. Kurata JH, Marks J, Abbey D. One gram of aspirin per day does not reduce risk of hospitalization for gallstone disease. Dig Dis Sci 1991;36:1110-5.

49. Ashley MJ. Smoking and diseases of the gastrointestinal system: an epidemiological review with special reference to sex differences. Can J Gastroenterol 1997;11:345-52.

50. Katsika D, Tuvblad C, Einarsson C, Lichtenstein P, Marschall HU. Body mass index, alcohol, tobacco and symptomatic gallstone disease: a Swedish twin study. J Intern Med 2007;262:581-7.

51. Halldestam I, Kullman E, Borch K. Incidence of and potential risk factors for gallstone disease in a general population sample. Br J Surg 2009;96:1315-22.

Supplementary Figure 1. Unclear smoking status and gallbladder disease

Supplementary Figure 2. Funnel plot of current smoking and gallbladder disease

Supplementary Figure 3. Funnel plot of former smoking and gallbladder disease

Supplementary Figure 4. Funnel plot of former smoking and gallbladder disease

Supplementary Figure 5. Influence analysis of current versus never smokers and gallbladder disease

------------------------------------------------------------------------------

Study omitted | e^coef. [95% Conf. Interval]

-------------------+----------------------------------------------------------

Liu, 2009 | 1.1900235 1.0754509 1.3168021

Gonzalez-Perez, 2007| 1.2176288 1.1816623 1.2546902

Sahi, 1998 | 1.1800362 1.1078725 1.2569004

Grodstein, 1994 | 1.1878734 1.1046557 1.27736

Kato, 1992 | 1.1863183 1.1026666 1.276316

Stampfer, 1992 | 1.2115966 1.1015738 1.3326081

-------------------+----------------------------------------------------------

Combined | 1.1933112 1.1168182 1.2750433

------------------------------------------------------------------------------

Supplementary Figure 6. Influence analysis of former versus never smokers and gallbladder disease

------------------------------------------------------------------------------

Study omitted | e^coef. [95% Conf. Interval]

-------------------+----------------------------------------------------------

Liu, 2009 | 1.0973573 1.0162868 1.1848947

Gonzalez-Perez, 2007| 1.0973872 1.0650233 1.1307346

Sahi, 1998 | 1.098515 1.0664628 1.1315306

Grodstein, 1994 | 1.0996044 1.0674466 1.132731

Kato, 1992 | 1.0996035 1.06741 1.1327679

Stampfer, 1992 | 1.1035589 1.0699544 1.1382188

-------------------+----------------------------------------------------------

Combined | 1.0996088 1.0676276 1.1325479

------------------------------------------------------------------------------

Supplementary Figure 7. Influence analysis of ever versus never smokers and gallbladder disease

------------------------------------------------------------------------------

Study omitted | e^coef. [95% Conf. Interval]

-------------------+----------------------------------------------------------

Liu, 2009 | 1.1442467 1.0876533 1.2037847

Gonzalez-Perez, 2007| 1.158832 1.1323466 1.1859368

Yamada, 2005 | 1.151273 1.1144116 1.1893537

Sahi, 1998 | 1.1525278 1.1267693 1.1788751

Grodstein, 1994 | 1.1515621 1.1152484 1.1890581

Kato, 1992 | 1.1513075 1.1151527 1.1886343

Stampfer, 1992 | 1.1602341 1.1326205 1.1885209

-------------------+----------------------------------------------------------

Combined | 1.1549623 1.1292883 1.1812201

------------------------------------------------------------------------------

Supplementary Figure 8. Cumulative meta-analysis of current smoking and gallbladder disease

Supplementary Figure 9. Cumulative meta-analysis of former smoking and gallbladder disease

Supplementary Figure 10. Cumulative meta-analysis of ever smoking and gallbladder disease
